# Assemblages of terrestrial isopods (Isopoda, Oniscidea) in a fragmented forest landscape in Central Europe

**DOI:** 10.3897/zookeys.176.2296

**Published:** 2012-03-20

**Authors:** Karel Tajovský, Jan Hošek, Jeňýk Hofmeister, Jolanta Wytwer

**Affiliations:** 1 Institute of Soil Biology, Biology Centre of the Academy of Sciences of the Czech Republic, Na Sádkách 7, 370 05 České Budějovice, Czech Republic; 2 Ecological Services Ltd., Areál ČOV, 268 01 Hořovice, Czech Republic; 3 Museum and Institute of Zoology, Polish Academy of Sciences, Wilcza 64, 00-679 Warszawa, Poland

**Keywords:** Woodlice, densities, epigeic activity, pitfall trapping, *Armadillidium vulgare*, *Porcellium collicola*

## Abstract

Terrestrial isopods were collected in 13 forest fragments differing in area (within the range of 0.1 and 254.5 ha), shape and composition of forest vegetation (thermophilous oak, mesophilous oak-hornbeam, thermophilous oak-hornbeam, acidophilous oak, basiphilous oak, beech oak-hornbeam, moist mixed deciduous forest, plantations of deciduous and coniferous trees), all situated in the Český kras Protected Landscape Area, Czech Republic, Central Europe. Number of sites sampled in each fragment of forest depended on its size and ranged from 1 to 7. Altogether 30 sites were sampled. Soil samples (5 per site collected twice a year) and pitfall trapping (5 traps per site in continuous operation throughout a year) during 2008–2009 yielded a total of 14 species of terrestrial isopods. The highest densities and highest epigeic activities of terrestrial isopods were recorded in the smallest fragments of woodland. Although a wider range of habitats were sampled in the larger fragments of woodland there was not a greater diversity of species there and the population densities and epigeic activities recorded there were lower. *Porcellium collicola* was most abundant in small fragments of woodland regardless the vegetation there. *Armadillidium vulgare* and *Protracheoniscus politus* were statistically more abundant in the larger fragments of woodland. The results indicate that forest fragmentation does not necessarily result in a decrease in the species richness of the isopod assemblages in such habitats.

## Introduction

Fragmentation is a natural feature of most landscapes (Tews et al. 2004). In addition to the possible decrease in biodiversity due to loss of habitats and extinction of sensitive species (Henle et al. 2004), habitat fragmentation has weaker positive than negative effects on biodiversity (Fahrig 2003). Invertebrates may react to habitat fragmentation differently (Ewers and Didham 2006) as it can positively influence the development and diversification of habitats and more-specialized species may be more susceptible to these processes than generalists (Didham et al. 1996).

The Central European landscape has been influenced by human activities for millennia. It is characterised by intensive land-use and a continuing trend of habitat destruction. Originally the landscape in the temperate zone was covered with woodland. Present forest system consists of patches differing in shape, size and vegetation in which there is often a higher biodiversity than in the majority of open and usually agriculturally exploited areas. Fragmentation of remaining areas of natural, semi-natural and other well-preserved patches of forest represents a major threat to biodiversity (Tscharntke et al. 2002). However, it is unknown whether such processes also influence the diversity of soil invertebrates (Didham et al. 1996). Although some studies suggest that soil organisms, in general, are not sensitive to habitat fragmentation, even at a small scale (Rantalainen et al. 2008, David and Handa 2010), this is not the case for rare, more specialised species or those with a poor dispersal ability (Tscharntke et al. 2002).

In the assessment of the effect of forest fragmentation on vegetation and different groups of aboveground and soil invertebrates, terrestrial isopods are used as a model group of soil saprophagous macro-invertebrates. Terrestrial isopods are also potential bio-indicators of environmental quality in natural as well as disturbed and polluted habitats (Dallinger et al. 1992, Paoletti and Hassall 1999). Presented in this paper is a comparison of the assemblages of terrestrial isopods in forest fragments differing in area and other structural parameters. The aim of the paper is to assess how the above (epigeic activity) and belowground (soil) parts of assemblages of terrestrial isopods react to forest fragmentation.

## Materials and methods

This research was undertaken in the fragmented landscape of the Český kras Protected Landscape Area, Central Bohemia, Czech Republic. In this area the bedrock is predominantly limestone and there are numerous fragments of formerly more integrated woodlands. There is little diversity in the structure of the vegetation in the smaller fragments, which contrasts with the mosaic character and higher spectrum of plant associations with a long history of diverse management and development in the larger forest units. During the period 2008-2009 soil sampling and pitfall trapping were used to determine the assemblages of terrestrial isopods in 13 fragments of forest that ranged in area between 0.1 and 254 ha. A total of 30 sites were sampled (Table 1). The sites sampled were representative of the seven forest plant associations characteristic of the area: thermophilous oak (TO), mesophilous oak-hornbeam (MOH), thermophilous oak-hornbeam (TOH), acidophilous oak (AO), basiphilous oak (BO), beech oak-hornbeam (BOH), moist mixed deciduous forest (MDF) and plantations of deciduous (DP) and coniferous (CP) trees. At each site, five soil samples (area of each 625 cm^2^, depth ca 10 cm) were collected in spring and autumn and isopods were heat extracted using a modified Kempson apparatus (Kempson et al. 1963). In addition, five pitfall traps (each with a diameter of 9 cm and containing a solution of formaldehyde) were used to catch isopods, which were collected from the traps once a month for a period of year at each study site. Additional soil samples were collected at each site and used to determine the chemical characteristics of the uppermost soil layers (soil pH, C:N ratio and Ca^2+^ content).

**Table 1.** The species of terrestrial isopods present at the different sites studied recorded in (▲) pitfall trap catches, (▼) extracts of soil samples and (+) by both methods. Total number of each species collected at each site in each fragment of woodland, frequency of occurrence of individual species (F) in % and abbreviations of species names used in the statistical analyses.

**Fragment of woodland (ha)**	0.1	0.3	0.8	4.5	9.5	11.2		13.6		14.2		15.3	18.9				20.9					35.9		254.5							
**Sites**	1	2	3	4	5	6	7	8	9	10	11	12	13	14	15	16	17	18	19	20	21	22	23	24	25	26	27	28	29	30	
**Vegetation**	BO	TO	DP	TO	MOH	DP	AO	MOH	MDF	AO	AO	MOH	TOH	TOH	MOH	DP	MOH	BO	CP	TO	BO	TOH	TOH	CP	TO	BOH	TO	TO	AO	MOH	F (%)
*Armadillidium vulgare* (Latreille, 1804) Armvul	+	+	+	+	+	+	+	+	▲	+	+	+	+	+	+	+	▲	+	+	+	+	+	+	+	+	▲	+	+	+	+	100.0
*Cylisticus convexus* (De Geer, 1778) Cylcon	▲	-	-	-	-	▲	▲	-	-	-	-	-	-	-	-	-	-	-	▲	-	-	-	-	▲	-	-	-	-	-	-	16.7
*Haplophthalmus mengii* (Zaddach, 1844) Hapmen	-	-	-	-	-	-	-	-	-	-	-	-	-	-	-	-	-	-	-	-	-	-	-	-	-	+	-	-	-	-	3.3
*Hyloniscus riparius* (C.L.Koch, 1838) Hylrip	-	-	▲	-	-	▲	▲	▲	▲	-	-	-	-	-	-	-	-	-	-	-	-	-	-	-	-	-	-	-	-	-	16.7
*Lepidoniscus minutus* (C.L.Koch,1838) Lepmin	-	-	-	-	-	-	-	-	-	-	-	-	-	-	-	-	-	-	-	-	-	-	-	-	-	▲	-	-	-	-	3.3
*Platyarthrus hoffmannseggii* Brandt, 1833 Plahof	-	-	▲	-	-	▲	▲	-	-	-	-	-	-	▲	▼	-	-	▲	-	-	-	-	-	-	-	-	-	-	-	-	20.0
*Porcellio scaber* Latreillle, 1804 Porsca	-	-	-	-	-	▲	-	-	-	-	-	-	-	-	-	-	-	-	-	-	-	-	-	-	-	-	-	-	-	-	3.3
*Porcellionides pruinosus* (Brandt, 1833) Porpru	▲	-	-	-	-	-	-	-	-	-	-	-	-	-	-	-	-	-	-	-	-	-	-	-	-	-	-	-	-	-	3.3
*Porcellium collicola* (Verhoeff, 1907) Porcol	+	+	+	+	+	+	+	+	+	+	+	-	+	+	+	▲	▲	▲	▲	+	▲	-	+	▲	-	+		+	+	▼	86.7
*Protracheoniscus politus* (C.Koch, 1841) Propol	+	+	-	+	+	-	-	+	-	+	+	+	+	▲	+	+	+	▲	+	+	+	+	+	+	+	+	+	+	+	+	86.7
*Trachelipus nodulosus* (C.Koch, 1838) Tranod	-	-	-	-	-	-	▲	-	-	-	-	-	-	-	▲	-	-	-	-	-	-	-	-	-	-	-	-	-	-	-	6.7
*Trachelipus rathkii* (Brandt, 1833) Trarat	-	▲	-	-	-	▲	-	-	-	-	-	-	-	-	-	-	-	-	-	-	-	-	-	-	-	-	-	-	-	-	6.7
*Trachelipus ratzeburgii* (Brandt, 1833) Traraz	-	-	▲	+	+	▲	-	-	▲	▲	▲	▲	▲	-	▲	▲	▲	-	+	+	▲	▲	▲	▲	▲	▲	▲	▲	▲	▲	80.0
*Trichoniscus pusillus* Brandt, 1833 Tripus	-	-	-	-	-	-	-	▼	+	-	-	-	-	-	-	-	-	▼	-	-	-	-	-	-	-	▲	▲	-	-	-	16.7
**Total number of spp.**	5	4	5	4	4	8	6	5	5	4	4	3	4	4	6	4	4	5	5	4	4	3	4	5	3	7	4	4	4	4	
**Species per fragment**	5	4	5	4	4	9		8		4		3	6				7					4		8							

The data on the isopod assemblages were evaluated with respect to the parameters of the sites and fragments. Multivariate analyses: DCA and RDA were used to consider gradient data length and importance of factors shaping the terrestrial isopod assemblages (13 variables). Methods of material collection (pitfall traps and soil samples) were treated as covariables in block analysis. The importance of the explanatory variables considered was examined during forward selection procedure in RDA. The analyses were done using the software for canonical community ordination – Canoco 4.5/CanoDraw 4.14 (ter Braak and Šmilauer 2002).

## Results

During the course of the two years of this study we collected 28 thousand isopods (1,839 were extracted from soil samples and 26,398 were caught in the pitfall traps) belonging to 14 species (Table 1). The most abundant of which were *Armadillidium vulgare*, *Porcellium collicola* and *Protracheoniscus politus*. The first species was recorded at all the sites and the frequency of occurrence of the other two species was 86.7 %. High frequency of occurrence (80.0 %) was recorded also for *Trachelipus ratzeburgii*. Whereas the first three species were generally predominant in both the pitfall trap catches and soil extracts *Trachelipus ratzeburgii* mainly inhabits aboveground microhabitats and is frequently found under the bark of decaying wood. Thus, it was repeatedly caught in pitfall traps, but often absent in extracts of soil samples.

Individual sites were frequently inhabited by only four or five species. The highest number of species was found in both small and large fragments (9 and 8 species in fragments of 11.2 and 254.5 ha, respectively).

**Figure 1.**
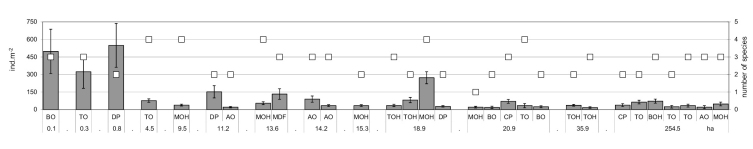
Total population densities (ind.m-2 ± SE) (columns) and numbers of species (white squares) in the isopod assemblages at the sites sampled in the different fragments of woodland.

**Figure 2.**
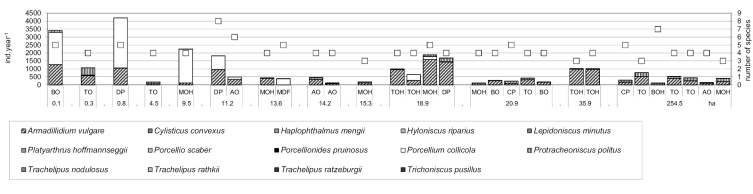
Epigeic activity (columns) (total catch per 5 traps per year) and numbers of species (white squares) of isopods at the sites studied in the different fragments of woodland.

Even the smallest fragments, which varied little in the structure of their vegetation, harboured high population densities of a high number of species. Both, the highest population density (549 individuals per m^2^) and epigeic activity (4,206 individuals per 5 traps per year) were recorded in the smallest fragments (Figure 1 and 2). In spite of sampling a greater number of sites in the medium-sized and large fragments of forest the lowest population densities and epigeic activities were recorded there.

**Figure 3.**
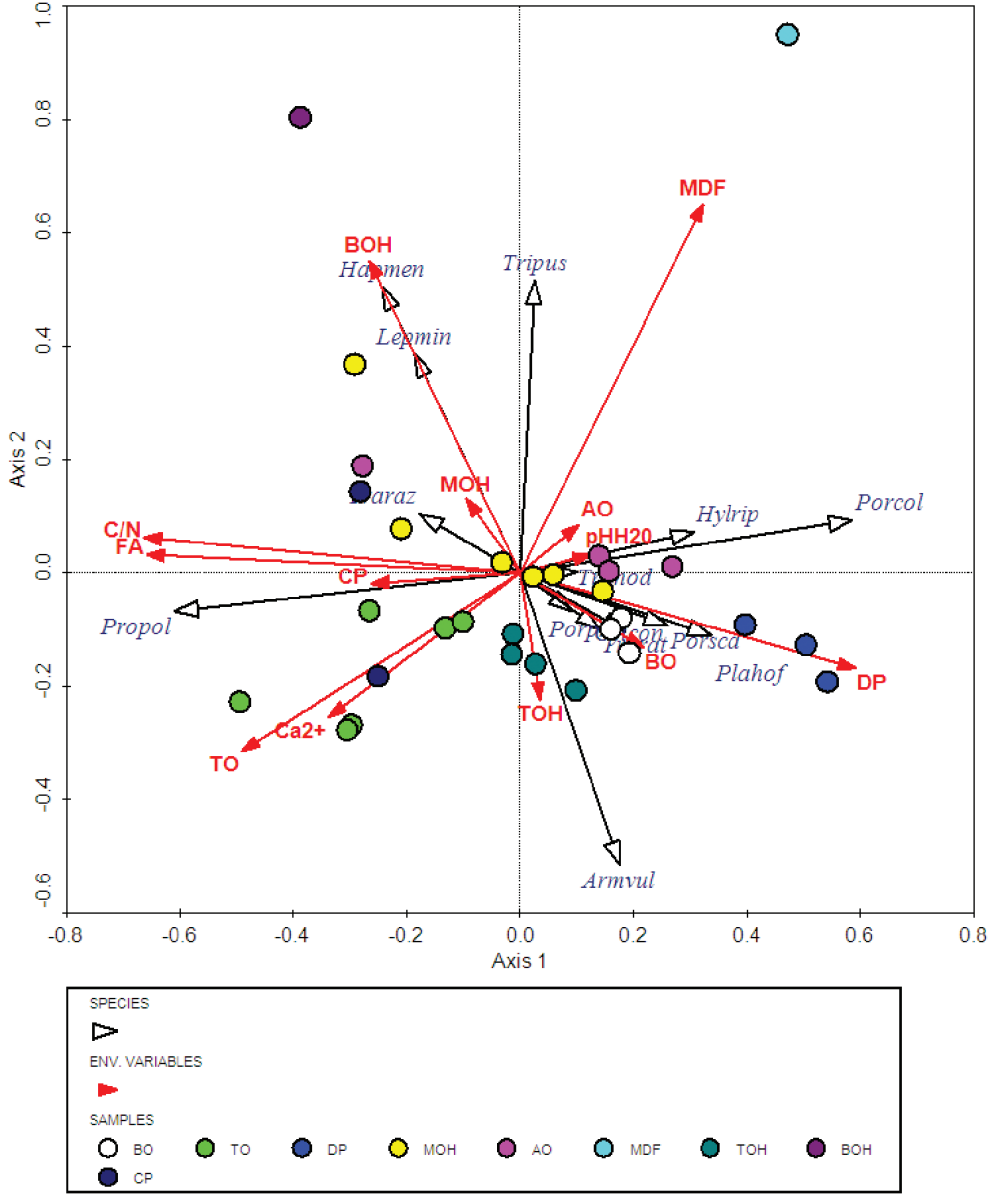
A triplot of the RDA block analyses of terrestrial isopod assemblages at the sites studied. For the abbreviations of the species see Table 1, FA – fragment area, C/N – carbon-nitrogen ratio, Ca2+ – calcium content of the soil, pHH2O – soil acidity. TO, MOH, TOH, AO, BO, BOH, MDF, DP and CP – vegetation at the different sites, see text.

Since the weighted-average Detrended Correspondence Analysis (DCA) indicates that isopods have a rather low beta-diversity, i.e. a ‘short gradient’ (=2.265), where most species show a linear response to the explanatory variables considered (Leps and Smilauer 2003), a triplot of the Redundancy Analysis (RDA) is presented (Figure 3). This indicates that the 1^st^ canonical axis is correlated negatively mainly with the area of the fragments (FA) and C:N ratio (Figure 3). Most species are significantly positively correlated with the 1^st^ axis. *Protracheoniscus politus* is correlated positively with size of a fragment and the C:N ratio whereas the second most abundant species, *Porcellium collicola*, is negatively correlated with these variables. The abundance of *Armadillidium vulgare*, which was abundant at most sites, was not associated with either FA or the C:N ratio. The two relatively rare species *Haplophthalmus mengii* and *Lepidoniscus minutus* were mainly associated with beech oak-hornbeam sites (BOH) and low population densities of the hygrophilous *Trichoniscus pusillus* and *Hyloniscus riparius* partly with moist deciduous forest.

The forward selection procedure revealed that the area of a fragment was the most important significant variable (marginal effect: λ1 = 0.1) determining the assemblage of terrestrial isopods (Table 2). The C:N ratio was highly correlated with FA and also may influence the variability in terrestrial isopod assemblages (marginal effect: λ1 = 0.1), however this association is not statistically significant.

**Table 2.** The results of the verification of the explanatory variables using the Monte Carlo permutation test (499 permutations under reduced model) in the forward selection procedure of RDA (CANOCO 4.5); significant variables are in bold.

**Marginal Effects**			**Conditional Effects**				
Variable	Var.N	Lambda1	Variable	Var.N	LambdaA	P	F
**FA**	**4**	**0.10**	**FA**	**4**	**0.10**	**0.002**	**8.51**
C:N	3	0.10	**MDF**	**10**	**0.06**	**0.002**	**4.94**
**DP**	**7**	**0.08**	**DP**	**7**	**0.05**	**0.008**	**5.53**
TO	6	0.07	**BOH**	**13**	**0.04**	**0.020**	**3.60**
**MDF**	**10**	**0.06**	TO	6	0.02	0.076	2.45
**BOH**	**13**	**0.05**	MOH	8	0.01	0.264	1.22
Ca2+	2	0.03	CP	12	0.02	0.192	1.48
CP	12	0.02	TOH	11	0	0.440	0.83
BO	5	0.02	C/N	3	0.01	0.398	1.00
TOH	11	0.01	BO	5	0.02	0.144	1.82
MOH	8	0.01	Ca2+	2	0.02	0.158	1.65
pHH_2_0	1	0.01	pHH_2_0	1	0	0.962	0.17
AO	9	0					

## Discussion

The three predominant species are differently associated with the area (FA), C:N ratio, soil acidity (pH) and Ca2+ content of the soil in the fragments of woodland studied. The isopods *Armadillidium vulgare* and *Porcellium collicola* were more abundant in small fragments of woodland. Nevertheless, *Armadillidium vulgare* was frequently recorded in the largest fragments. *Protracheoniscus politus* was most abundant in large fragments of woodland. The RDA also reveals a close association of *Armadillidium vulgare* and *Porcellium collicola* with fragments of woodland with base rich soils (high pH), whereas for *Protracheoniscus politus* this association is less pronounced. Some species (e.g. *Cylisticus convexus*, *Trachelipus ratzeburgii*, *Porcellio scaber*, *Trachelipus nodulosus* and *Trachelipus rathkii*) may be more acid tolerant.

Although *Armadillidium vulgare* was abundant at most of the sites studied, both in small and large fragments of woodland (see Figure 2), *Porcellium collicola* was most abundant in the small fragments of woodland irrespective of the vegetation at these sites. The third most frequent species, *Protracheoniscus politus*, was most closely associated with large fragments of woodland. Its occurrence in some of the smaller fragments may be attributed to the historical fact that mainly due to man the forest in this area was fragmented into small separate wooded islands during the course of the past century.

The isopod communities in the larger fragments of forest, which have the greatest diversity of habitats, were the most homogeneous. Surrounding open grassland and forest-steppe calcareous biotopes, including diverse man-made habitats, did not enrich the diversity of isopods recorded in forest fragments as synanthropic species were always in the minority.

The abundance and composition of the species in isopod assemblages differed depending on the plant associations in the fragments of woodland sampled, but in vegetation types TO, TOH and AO they were very similar.

Terrestrial isopods do not appear to be more sensitive to fragmentation than other saprophagous invertebrates, such as millipedes (David and Handa 2010). The fragmentation of shrubby habitats in urban areas does not reduce the epigeic activity of *Armadillidium vulgare* and *Porcellio laevis* (Bolger et al. 2000). Apparently the critical fragment size for these animals is very small or they are better at dispersing than generally thought (David and Handa 2010).

Our results indicate that forest fragmentation does not necessarily result in a decrease in the size of terrestrial isopod assemblages, but their dominance structure may be affected, which is in accordance with the results presented in the David and Handa’s (2010) review. The determination of the sensitivity of different species to fragmentation is dependent on further analyses of the changes in the population parameters associated with other environmental characteristics that occur following fragmentation.
